# Supplementary figures and images for: Systematic analysis of SARS-CoV-2 Omicron subvariants’ impact on B and T cell epitopes
Source: PLoS One. 2024 Sep 19;19(9):e0307873. doi: 10.1371/journal.pone.0307873 (PMC11412522; doi:10.1371/journal.pone.0307873)

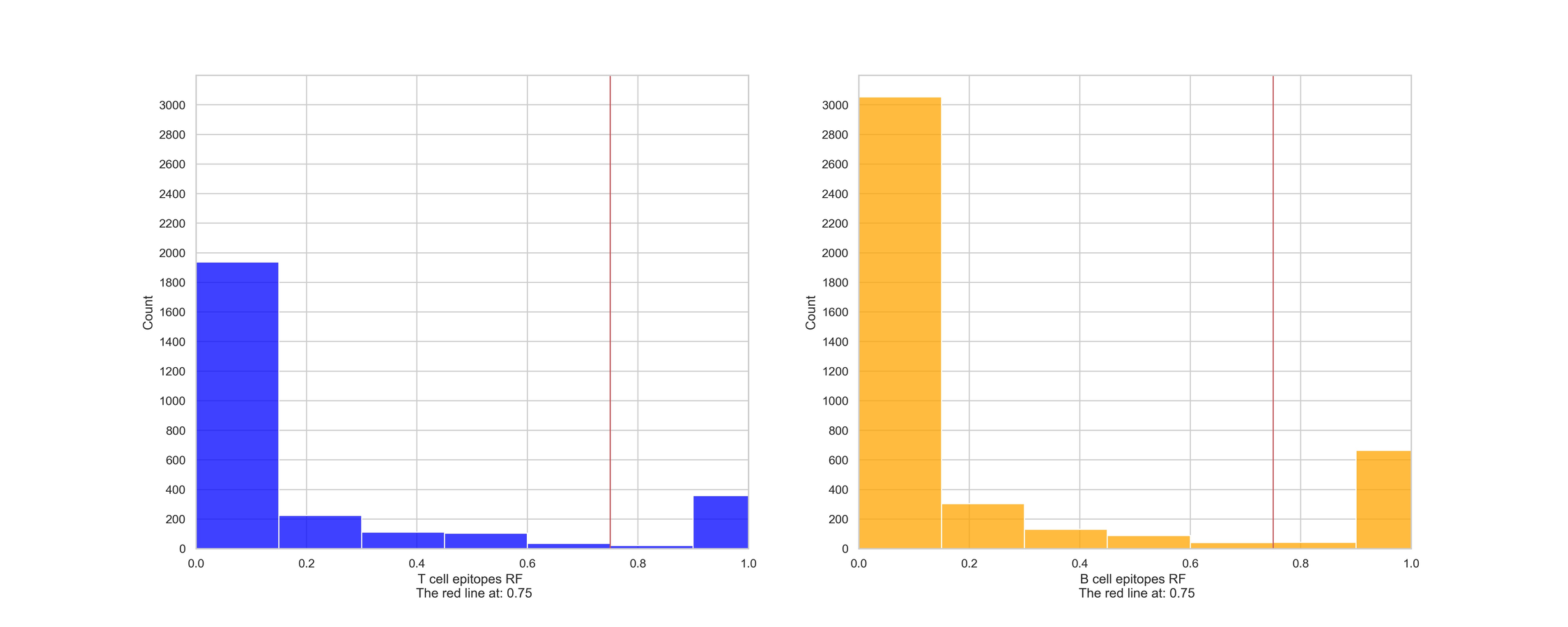

Supplement: S1 Fig — The vertical red line (RF = 0.75) is the selected threshold for filtering both cases. (TIF) [file pone.0307873.s001.tif]

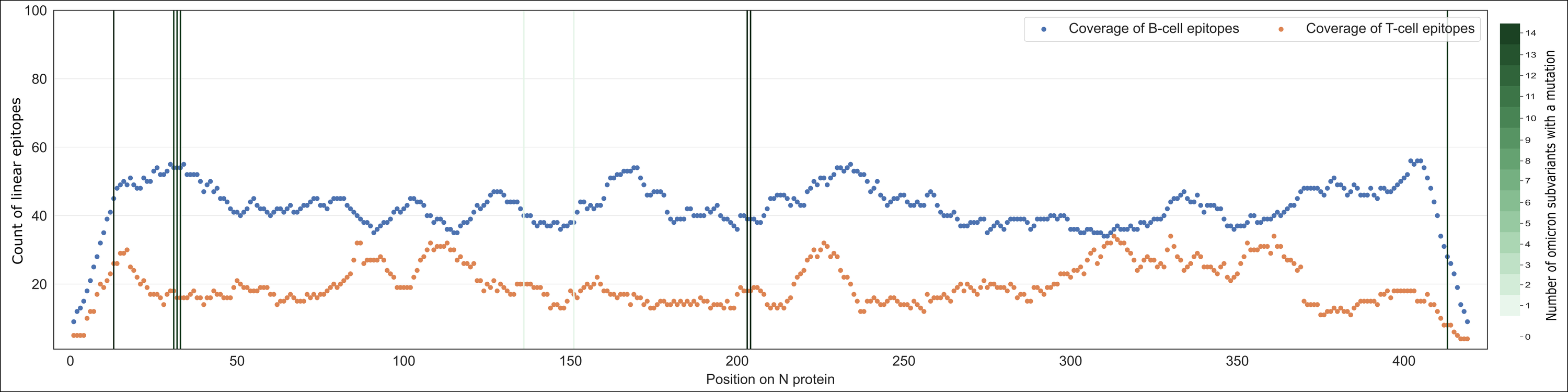

Supplement: S2 Fig — Vertical lines indicate positions of mutations from Mv for all v ∈ OV; the darker the green color, the higher the number of variants with a mutation at that position. (TIF) [file pone.0307873.s002.tif]

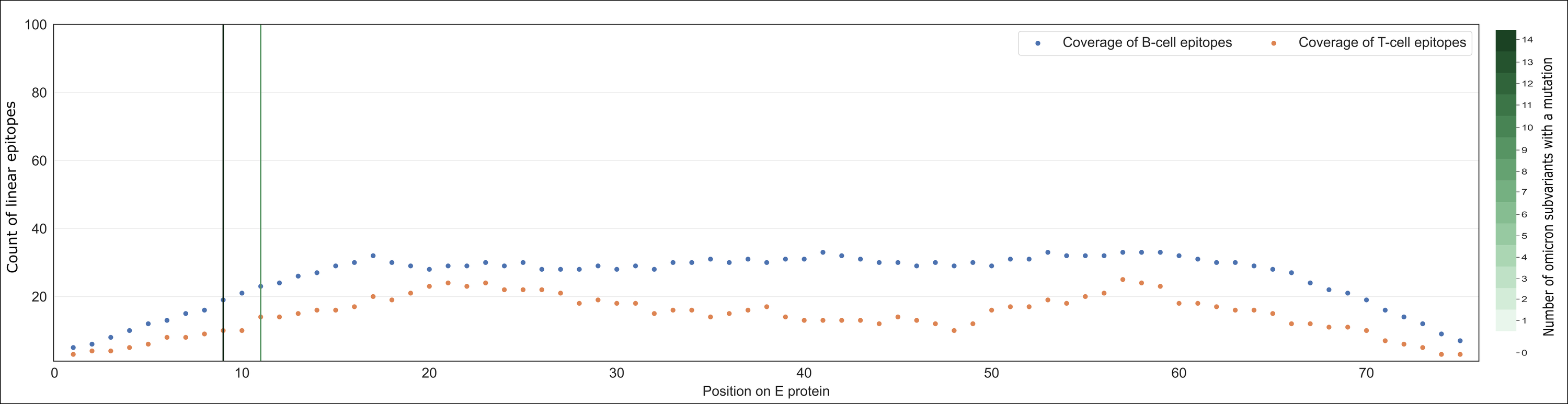

Supplement: S3 Fig — Vertical lines indicate positions of mutations from Mv for all v ∈ OV; the darker the green color, the higher the number of variants with a mutation at that position. (TIF) [file pone.0307873.s003.tif]

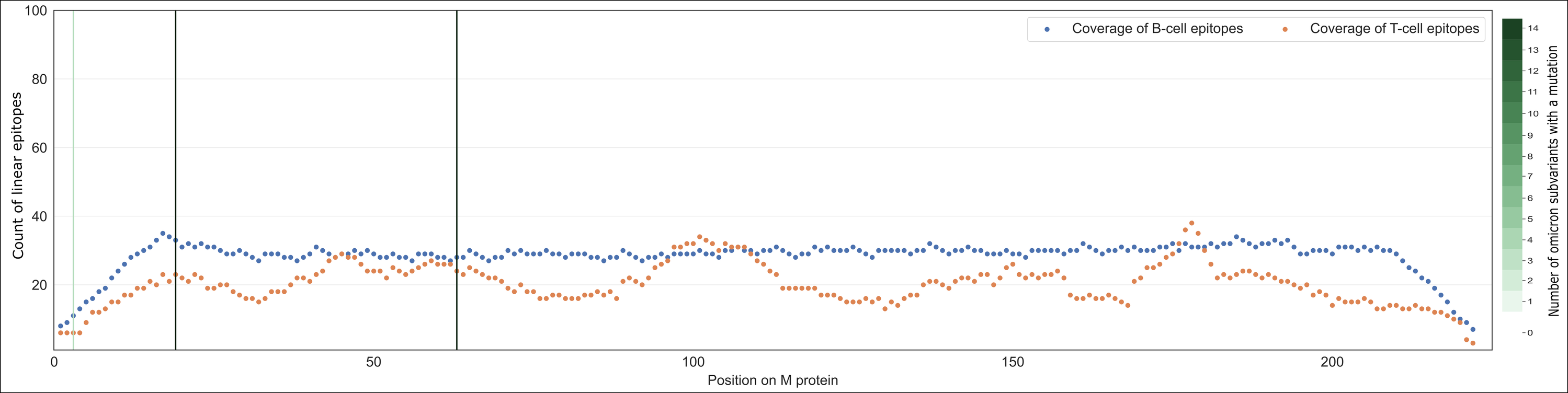

Supplement: S4 Fig — Vertical lines indicate positions of mutations from Mv for all v ∈ OV; the darker the green color, the higher the number of variants with a mutation at that position. (TIF) [file pone.0307873.s004.tif]

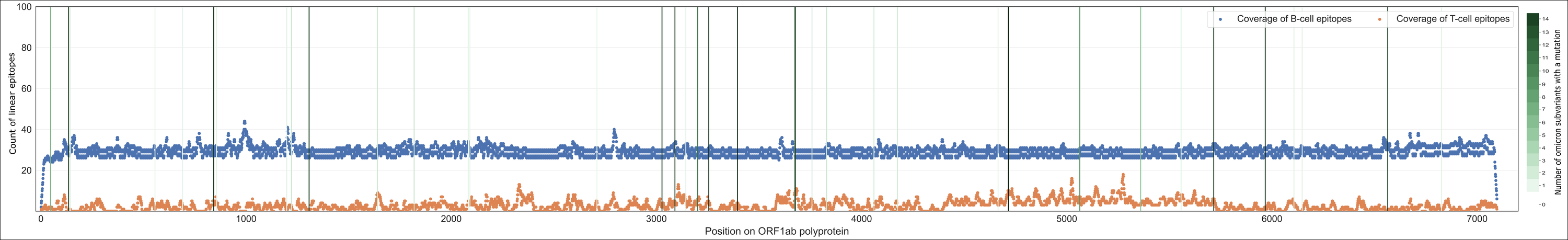

Supplement: S5 Fig — Vertical lines indicate positions of mutations from Mv for all v ∈ OV; the darker the green color, the higher the number of variants with a mutation at that position. (TIF) [file pone.0307873.s005.tif]

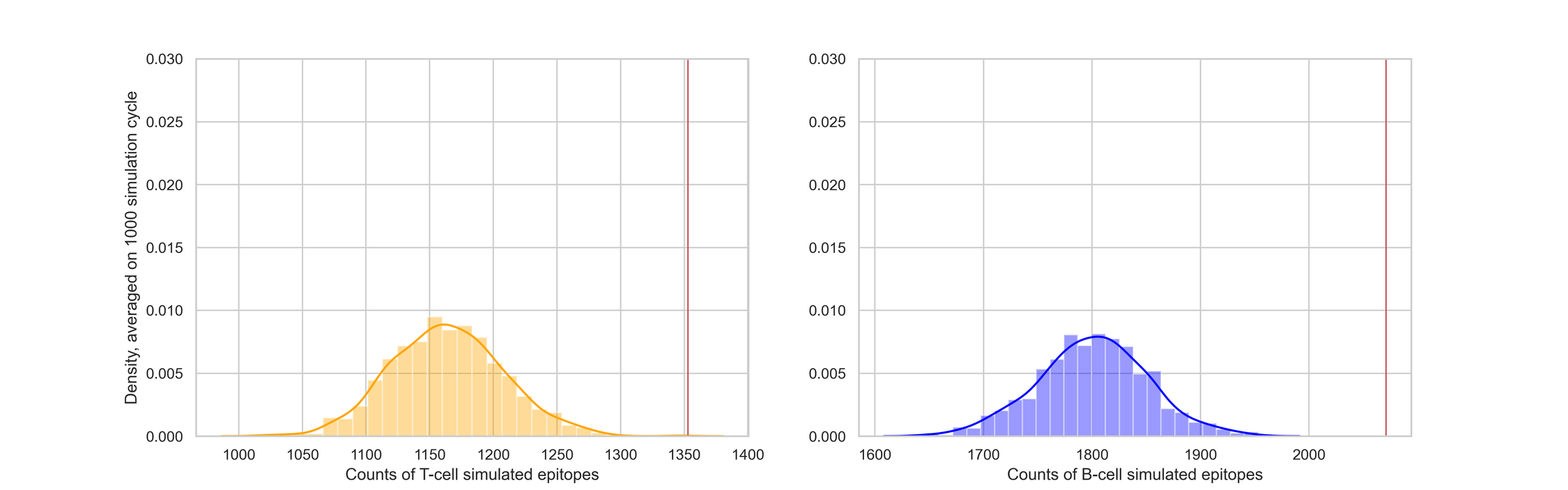

Supplement: S6 Fig — We plot the density distribution of T-cell (left) or B-cell (right) simulated epitopes’ factors (see Methods for details), affected by at least one sampled mutation. The red vertical lines (at 1353 T cell and 2071 B cell epitopes) represent the observed sum of the computed factors of all linear epitopes from the Spike protein that have been mutated. (TIF) [file pone.0307873.s006.tif]

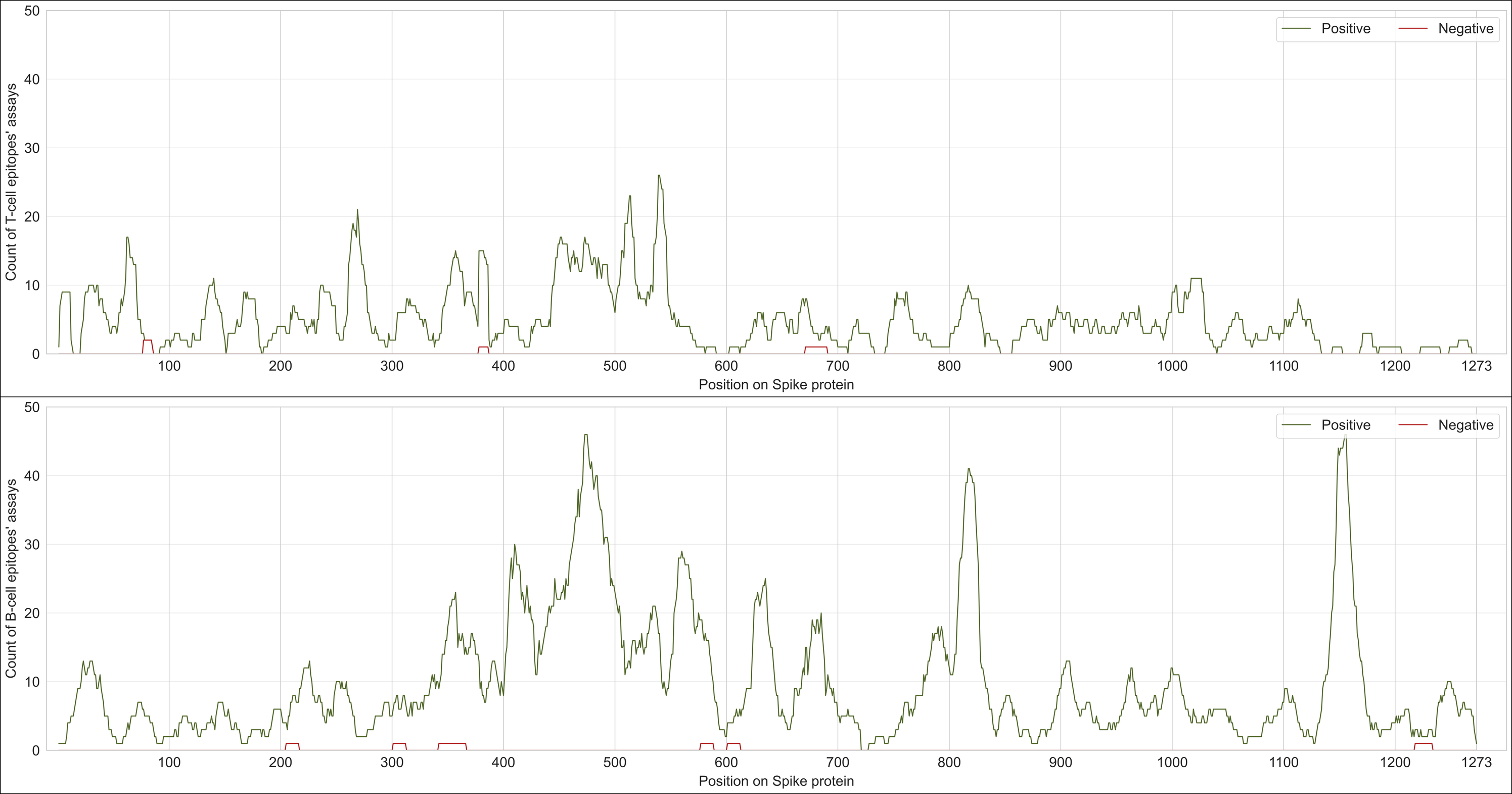

Supplement: S7 Fig — (TIF) [file pone.0307873.s007.tif]

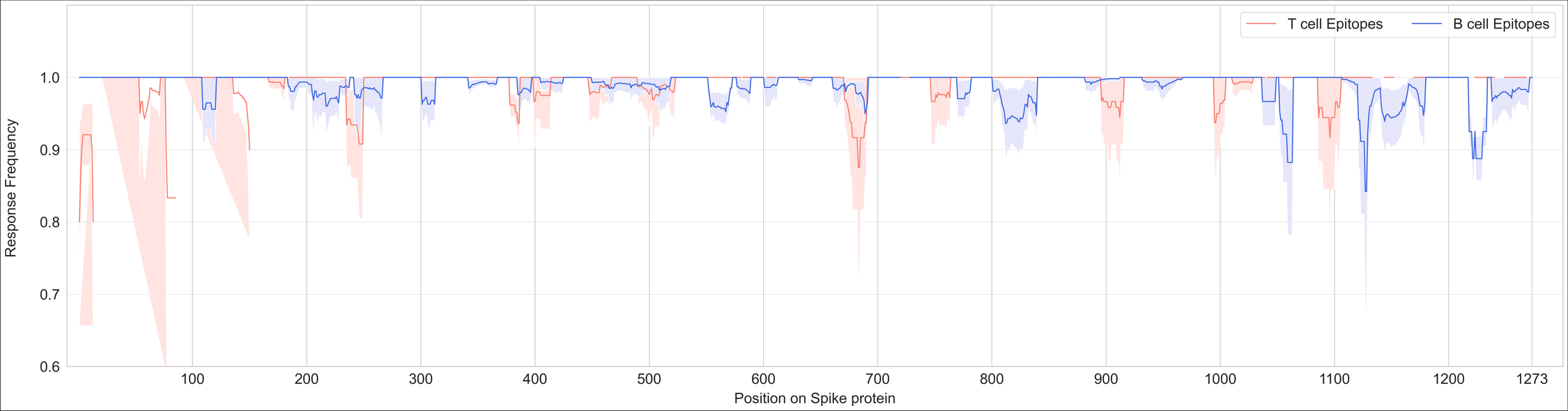

Supplement: S8 Fig — (TIF) [file pone.0307873.s008.tif]

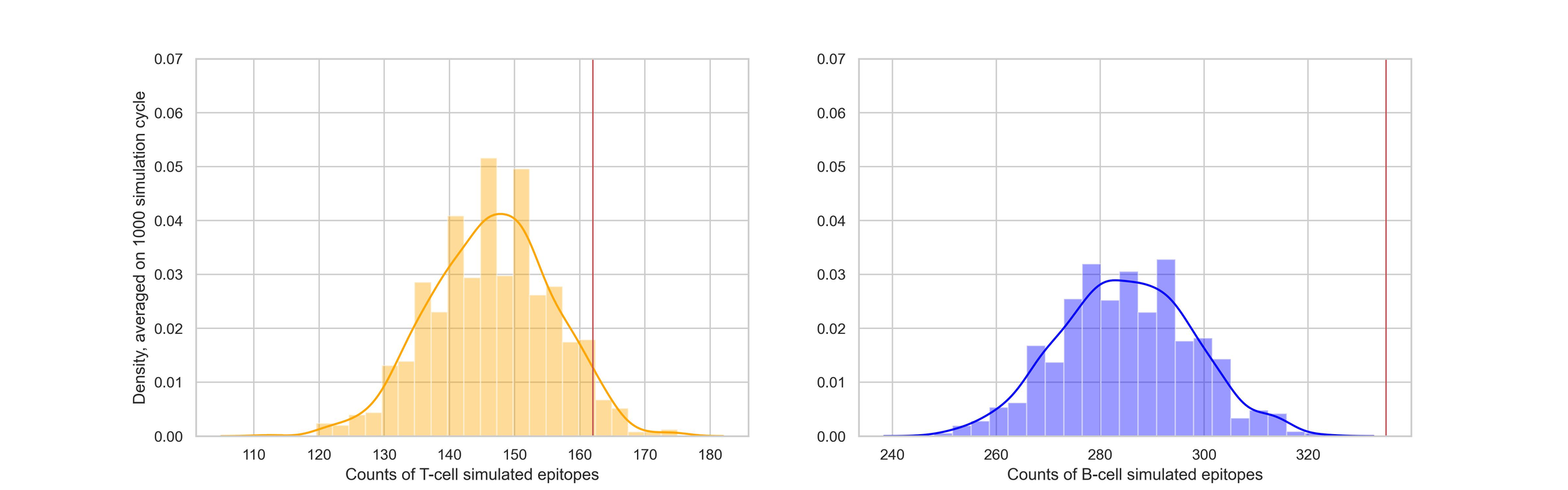

Supplement: S9 Fig — We plot the density distribution of T-cell (left) or B-cell (right) simulated epitopes affected by at least one sampled mutation. The red vertical lines (at 162 T cell and 335 B cell epitopes) represent the observed count of linear epitopes from the epitopes in the selected subset of Spike protein that have been mutated. (TIF) [file pone.0307873.s009.tif]
