# Supplementary material for: Systematic analysis of SARS-CoV-2 Omicron subvariants’ impact on B and T cell epitopes
Source: PLoS One. 2024 Sep 19;19(9):e0307873. doi: 10.1371/journal.pone.0307873 (PMC11412522; doi:10.1371/journal.pone.0307873)
Supplement: Attachment — Submitted filename: Khalaf_et_al_reviewer_comments.docx [file pone.0307873.s013.docx]

The submitted research article by Khalaf et al. explores the localization of SARS-CoV-2 Omicron variant amino acid mutations within T cell and B cell epitopes collected in the Immune Epitope Database. The work leverages a data-science approach and provides a nice demonstration of analyzing and curating publicly available data. The analyses are appropriate and performed well, but some of their presentation led to confusion. I mark all these issues as ‘minor,’ because I don’t believe any new experiments or re-analysis will need to be performed. Nevertheless, I believe that modification/clarification of the text is critical for publication and to allow the hard work of this group to be clearly presented.

1. Figure legends need to be addressed. Figure legends should enable the figure to allow the reader to reasonably understand the data independent of the main text. Figure 9 is the biggest example. I will not clarify every point, but most figures (and supplementary figures) would benefit from improved description. A couple of specific points:
   a. Figure 8, also clarifying this was from simulated data (is it? See below), to prevent confusion with data from Figure 4.
   b. Figure 5 (and methodology/main text [Line 340]): what defines “considerable differences” that warrants highlighting
2. Calling the two different Monte Carlo methodologies “variants” is confusing, since variant is used so frequently used to refer to Omicron lineages. Would suggest finding another phrase to describe the two simulations.
3. Lines 391-400. I am confused by this section, and Figure 8. I can’t tell if this is ‘real’ data that has been included in the Monte Carlo simulation section, or simulated data that is not clearly described, or something else that I am misunderstanding. Is this data effectively the data from Figure 4, but using only RF>0.75 epitopes? I think the transition from simulated data to real data within the same section is creating some confusion.
4. If possible, including XY coordinate values of Figure 3 as an Excel/CSV file in the supplemental data may be helpful. This could allow researchers to quickly manipulate the data and find regions with high epitope coverage more easily.
5. Line 55: Change “contrast” to “combat”
